# Exploring the Operational Status and Challenges of Community-Based Mental Healthcare Centers in Taiwan: A Qualitative Analysis of Healthcare Professionals’ Insights

**DOI:** 10.3390/healthcare12010051

**Published:** 2023-12-25

**Authors:** Ching-Teng Yao, Hong Hong

**Affiliations:** 1 Master Program of Long-Term Care in Aging, Kaohsiung Medical University, Kaohsiung 80708, Taiwan; 2 Bachelor Program of Senior Health Promotion and Care Management for Indigenous People, National Changhua University of Education, Changhua 50007, Taiwan; oscarhong@cc.ncue.edu.tw

**Keywords:** community-based mental healthcare center, front-line worker, organizational analysis, operational status, operational difficulty

## Abstract

Psychological disorders have become more prevalent in the presence of modernization and societal changes. Community-based mental health is important in healthcare. Taiwan has passed the Mental Health Act, and county governments have established community-based mental healthcare centers. This study aimed to fill the research gap regarding the operational status of these centers. A qualitative study design using semi-structured interviews was used to obtain data from a purposive sample. Seventeen healthcare professionals who were front-line workers of a community-based mental healthcare center in Taiwan were interviewed individually. This study uses the organizational analysis structure as the research base. The data were analyzed using qualitative content analysis. The theme—“operational status and difficulties”—and two categories with twelve subcategories emerged. The findings demonstrate (1) unclear objectives and imprecisely defined roles, (2) incomplete services provided, an overly defined area, and ineffectiveness, (3) the central government lacking clear objectives and operational strategies, (4) the public being ignorant of mental diseases and the operation of the centers, and (5) the lack of local resources for mental and social welfare. The government should immediately form clear policies to improve community-based mental healthcare, clarify the structure and models, increase resources for the centers, and provide direct services.

## 1. Introduction

Mental illness ranks as the third highest contributor to the global burden of diseases, trailing behind only cardiovascular diseases and cancer [1]. In the United Kingdom, approximately 25% of the adult population annually experiences at least one mental health disorder [2]. In Taiwan, the 2018 survey by the Ministry of Health and Welfare disclosed that 8.9% of the population was affected by depression [3]. Furthermore, a 2016 report from the Ministry of Health and Welfare indicated that caregivers for individuals with chronic mental illnesses dedicated an average of 17.13 years to their caregiving roles [4]. Numerous studies have underlined the considerable negative effects of poor mental health on life expectancy, quality of life, and the risk of physical illnesses [5,6,7,8,9]. Global initiatives in public mental health are committed to enhancing psychological well-being and achieving equity in mental health across diverse populations, employing interdisciplinary approaches [10]. Traditionally, mental healthcare models prioritized treatment, confinement, and patient segregation. However, contemporary community-based mental health models place a strong emphasis on proactivity, prevention, and the promotion of both the mental and physical well-being of community members. They also embrace multiculturalism and recognize the profound influence of the social environment on individuals [11,12,13]. Consequently, community-based mental healthcare initiatives stress primary and secondary prevention to reduce the likelihood of mental illness occurrence and provide early interventions to mitigate the severity of mental health disorders [14,15]. Despite the proven cost-effectiveness of preventative measures for mental illnesses, the allocation of resources for mental health remains a relatively low priority in the healthcare sector [16]. Globally, fewer than 20% of individuals with mental disorders receive national resources for assistance [17].

In light of a series of recent incidents involving individuals with mental disorders causing harm within Taiwanese communities, it has become increasingly clear that the national social security response and coping mechanisms are insufficient. Acknowledging the challenges associated with caring for individuals with mental disorders, the Taiwanese government has launched the second phase of an initiative to bolster the social safety net. The objective is to ensure ongoing community care services for individuals with mental disorders when they reintegrate into the community after leaving the hospital. Consequently, in 2022, amendments were made to the Mental Health Act to appropriately address public concerns. The overall focus of these legislative amendments includes (1) advancing mental health promotion, (2) proactively establishing community-based mental healthcare centers and diversifying community support resources, (3) strengthening the reporting of individuals with mental disorders within the community and establishing crisis management protocols, and (4) enhancing the protection of the rights of individuals with mental disorders and preventing stigmatization [18,19].

Over the past three decades, Taiwan’s mental health policies have predominantly revolved around psychiatric treatment, often involving long-term institutionalization for those with mental illnesses. Substantial government funding has been allocated to establish psychiatric beds. Consequently, Taiwan has historically fallen short in its investments in community-based mental healthcare, resulting in inadequate support and limited family and social resources for individuals with mental illnesses upon their return to the community after hospitalization. This transition from the hospital to the community has often lacked continuity, with the community care system not being tailored to the needs of individuals with mental illnesses and their families. Consequently, family members bear the responsibility of long-term care for their loved ones with mental disorders without receiving sufficient assistance from national resources. In response to these challenges, the government has once again convened scholars, experts, mental health organizations, and family groups representing individuals with mental disorders to deliberate on the direction of community-based mental healthcare policies. The ultimate objective is to establish a comprehensive network of community-based mental healthcare centers, bolster individual case management for those with mental disorders, and enhance community care resources. Concurrently, there is an inclusive effort to promote the psychological well-being of the community. Nevertheless, it remains uncertain whether the recently introduced services of community-based mental healthcare centers in Taiwan have adequately addressed the mental health needs of the public, encompassing aspects such as improving the accessibility of community-based mental healthcare services and community care for individuals with mental disorders [20,21].

Community-based mental healthcare centers play a pivotal role in Taiwan’s mental health system and have garnered significant attention in recent key health policies set forth by the Ministry of Health and Welfare. In addition to the promotion of public mental health, these centers have been entrusted with the provision of community care resources for individuals with mental disorders and the delivery of home care and support to those who irregularly seek medical attention. The overarching aim is to strengthen primary prevention and mitigate criminal incidents stemming from mental health instability. In line with the government’s endeavors to implement the second phase of the social safety net project, a guiding principle has been established, mandating the establishment of one community-based mental healthcare center for every three to four townships, roughly equivalent to 330,000 residents. With this annual benchmark in mind, 28 community-based mental healthcare centers have been established by the conclusion of 2022 [22].

Despite the initiation of Taiwan’s community-based mental health system, only one article based on policy research on mental healthcare in Taiwan has been published [20]. For other countries, a search of the databases, CINAHL, EBSCO, and PubMed, for the period 2012–2022, found 32 articles in total, which were mainly about mental health recovery, rehabilitation, community psychiatry, mental health service, etc. [23], but qualitative interviews on community mental healthcare centers service were relatively rare. To deeply understand the operational status and the obstacles confronted by community mental healthcare centers in Taiwan, the aim of this study was to explore the experience of healthcare professionals to obtain insights into their community mental healthcare center service.

## 2. Methods

This study employed a qualitative research approach, primarily utilizing in-depth interviews to delve into the status quo and challenges associated with implementing community-based mental healthcare center services in Taiwan. The primary objective was to offer comprehensive recommendations that could enhance Taiwan’s community-based mental healthcare policies and service delivery. To underpin our research analysis, we drew upon the organizational theory analysis framework proposed by Wong and Millette [24]. Factors that impacted organizational operations were classified into two key dimensions, namely the internal organizational environment and the external environment, while decision-makers within the organization served as crucial agents within the operational processes of the organization. By exploring both internal and external factors of influence, the study aimed to gain a more nuanced understanding of the current status and challenges within the realm of community-based mental healthcare center services.

### 2.1. Participants

The study recruited healthcare professionals from 28 community-based mental healthcare centers located throughout Taiwan. A purposive sampling approach was utilized to extend invitations for in-depth interviews to either the directors or staff members of these community-based mental healthcare centers. The eligibility criteria were individuals who were employees of community-based mental healthcare centers at the time of the study, who had a minimum of one year of experience at their respective centers, and who expressed a wholehearted willingness to engage in comprehensive interviews. Those who fulfilled these selection criteria were invited to partake in the study. Each participant was provided with an explanation of the study’s background and objectives, and they all formally consented to participate in adherence to academic ethics. The research intervention spanned from March to December 2022. A total of 17 community-based mental healthcare center healthcare professionals were interviewed.

### 2.2. Research Ethics

This study was approved by the Institutional Review Board and was conducted with the consent of the institution. The researcher explained the objective and method of this study to the participants and acquired their written consent. Their anonymity and confidentiality were strictly protected. All research data were encoded to ensure the anonymity of the participants and used only for academic research purposes. The participants were permitted to withdraw from a session or to quit the study altogether during the research procedure for any reason.

### 2.3. Data Collection

This study utilized purposive sampling. The research team explained the study’s objectives to eligible participants. Once their consent was secured, one-on-one semi-structured interviews were carried out, with each interview lasting between 60 and 90 min and being recorded. Data collection persisted until content saturation was achieved. The interview questions centered on the practitioners’ implementation of community-based mental healthcare center services and the challenges they encountered in service delivery.

### 2.4. Data Analysis

The data collected underwent analysis using qualitative content analysis as outlined by Graneheim and Lundman [25]. This study adhered to the following steps: (1) The interview transcripts were meticulously read multiple times by the interviewee to establish a comprehensive grasp of the overall content. (2) The textual data were read and systematically coded, involving continual cross-referencing and comparison to unravel meanings and relationships within the data. (3) Inductive analysis was applied to the data to uncover shared themes, followed by classifying data with shared meanings and constructing core categories and subcategories. (4) Meanings, patterns, and concepts were extracted from the collected data, ultimately leading to the formulation of the study’s findings.

### 2.5. Rigor

We examined the rigor and trustworthiness of this study based on the four criteria proposed by Guba and Lincoln on the precision of qualitative research [26], as follows: (1) Credibility: The researchers had extensive experience in studying community-based mental health policies and were well acquainted with the process of promoting mental health policies in Taiwan. Furthermore, the researchers had received comprehensive training in qualitative research, demonstrating practical proficiency in conducting interviews and performing qualitative analysis. In addition, regular discussions with qualitative research experts were an integral part of the research process. (2) Transferability: Interviews were accurately and truthfully transcribed verbatim for presentation in this study. Transcription of interview content returned to participants for correction. (3) Dependability: We invited two community mental health professionals with extensive experience in qualitative research to review and modify the classification of the findings. (4) Conformability: The researchers safeguarded all the reflective field notes and records of data analysis in this study for future verification and reference. At the final stage of the study, the participants were given the opportunity to review and confirm the research outcomes.

## 3. Results

Seventeen participants were interviewed, comprising ten female and seven male participants. The average age was 35.7 years. Their professional backgrounds were primarily rooted in nursing, followed by social work. The majority of participants had accumulated 2–3 years of service (Table 1).

This study conducted interviews with 17 community-based mental healthcare center practitioners. An evaluation of the operational status of these centers encompassing organizational goals, human resources, operating budget, and professional expertise led to the identification of four categories. Additionally, an analysis of the operational challenges encountered by community-based mental healthcare centers was conducted through in-depth interviews with practitioners, resulting in the identification of eight categories (Table 2).

### 3.1. Operational Status of Community-Based Mental Healthcare Centers

#### 3.1.1. Organizational Goals

Nine centers primarily focused on resource referral rather than offering direct services. Typically, staff members assessed cases and, subsequently, referred them to appropriate organizations. The services primarily involved referring mental healthcare resources and providing informational materials. The centers’ organizational goals were predominantly geared toward resource referral to establish pertinent referral networks in order to aid individuals seeking assistance.

*Our center’s role is to act as a resource referral hub, and our goal is to connect with local mental healthcare resources and provide the public with relevant information. We do not have professional staff, and our operational model relies on integrated resource management and referral to provide services. The center does not engage in direct case services*.

(ST12)

*We are unable to provide case services; thus, our center’s stated goal in external promotion is to offer information about local mental healthcare resources in our city. For more serious cases, we assist in referring them to relevant organizations. Referral mainly involves informing the individual about available resources. In other words, we provide the individual with a resource list that we have compiled, followed by encouraging them to seek out these resources independently*.

(ST10)

*Currently, our capabilities are limited to resource networking and referrals. We do not offer individual case services. Instead, we refer such cases to hospitals, such as ×× Hospital’s psychiatric department or ×× nursing home*.

(ST1)

Additionally, interview data revealed that two of the community-based mental healthcare centers placed a significant emphasis on suicide prevention services. Their main organizational goals centered on suicide prevention, and their external promotional efforts prioritized reporting suicide cases, managing cases of suicide incidents, and advocating for suicide prevention.

*Our center primarily operates as the suicide reporting hub for ×× City. Whenever there are cases involving suspected high-risk groups or suicide incidents, the relevant authorities report to us. As a result, the center’s organizational goals lean significantly toward suicide prevention*.

(ST5)

*Our county currently has the highest suicide rate in Taiwan, which leads to significant attention from local authorities. Consequently, it has become the primary focus of the center’s operation, with the established goal being to reduce the suicide rate. Hence, the entire scope of our efforts currently revolves around suicide prevention*.

(ST9)

#### 3.1.2. Human Resources

The interview data demonstrated that two county governments placed significant importance on the development of community-based mental healthcare centers. Consequently, they hired full-time staff members to oversee operations, with backgrounds in psychology, social work, nursing, and related fields. However, it was also noted that in as many as nine centers, staffing was managed by one to two personnel from the health bureau who held multiple positions. This situation imposed limitations on the centers’ operational capacity.

*When the center was initially established, two counselors were recruited through open public recruitment. During that period, I, with a background in social work, and the other individual with a nursing background, both possessed professional expertise and a deep understanding of the center’s goals and operational mission, which greatly aided our pursuit of objectives*.

(ST16)

*Currently, community-based mental healthcare center operations are handled by personnel rotating from within the health bureau. This setup has been in place since the center’s inception. We had previously hired a temporary staff member, but eventually, the position was eliminated due to budget constraints*.

(ST10)

#### 3.1.3. Operational Budget

Eight centers primarily relied on subsidies from the Ministry of Health and Welfare, lacking their own sources of funding. In situations where the funding was insufficient, the centers were unable to fully utilize their service capabilities. However, it was also noted that three centers, in addition to subsidies from the Ministry of Health and Welfare, included a mental health budget allocated by the county or city government, as local leaders placed significant emphasis on this service.

*The operational budgets of the center mainly come from the Ministry of Health and Welfare. When the county lacks funds for a particular year, the center’s budget relies on the central government. This amount is limited and cannot adequately support the full functionality of the community-based mental healthcare center*.

(ST11)

*The center’s budget primarily comes from the Ministry of Health and Welfare (Laugh). It was only recently that they separated the budgets for psychiatric care and mental health. I can confidently say that the annual funding primarily depends on central government subsidies*.

(ST8)

*If the community-based mental healthcare center lacks resources, it cannot offer anything to the people. Our newly elected county mayor has a better understanding of this issue. Even though our ×× County does not have much money, we have allocated NT$600,000 from the social welfare budget to establish counseling services. Thankfully, these funds are pre-allocated*.

(ST7)

#### 3.1.4. Professional Expertise

The organization’s level of expertise significantly influences its functioning and service delivery. However, in up to nine community-based mental healthcare centers, staffing was primarily managed by personnel from the health bureau, who lacked relevant professional backgrounds. They had limited familiarity with mental healthcare services and frequently depended on experiential learning methods to perform relevant duties. The interviews revealed that the two centers better met the public’s professional expertise requirements. These two centers were considered more exemplary community-based mental healthcare centers, as they offered primary and secondary mental health prevention services to the public, surpassing the provision of mere information and referrals.

*In this area, there is not much professional expertise to speak of. From its establishment to the present, the center has not employed professionals with backgrounds in psychology or social work. So, it is challenging to claim any professional expertise. When cases require services, the center’s staff, lacking training in these fields, cannot effectively manage them, leaving them no option but to refer these cases elsewhere*.

(ST12)

*Providing direct services is crucial for the center. For instance, our psychological counseling services are delivered directly by the center’s psychologists. We follow an appointment-based system, and currently, if we hire additional counseling staff, we require them to hold licenses as psychologists or social workers. This not only ensures their competence for the center’s operation but also enables them to effectively offer direct services*.

(ST7)

### 3.2. Operational Challenges of Community-Based Mental Healthcare Centers

#### 3.2.1. Lack of Clarification in Organizational Goals and Role Definition

Organizational goals serve as the guiding principles and foundations for an organization’s activities. The research findings revealed a lack of specific planning for objectives in community-based mental healthcare centers, resulting in a vague definition of their roles in operations. This ambiguity created challenges in service implementation, with as many as eight community-based mental healthcare centers acknowledging this problem. Consequently, these centers faced severe criticism and operational constraints.

*From the outset, the organizational goals of the center have lacked clarity. This issue has persisted since the center’s establishment. The uncertainty surrounding the center’s objectives raises the fundamental question of whether it should primarily focus on delivering direct mental healthcare services that align with the public’s expectations or concentrate on resource referral activities*.

(ST4)

*There have been no discussions regarding the center’s operational objectives and its role, leaving the impression that it is merely another healthcare center with some budgetary support. This lack of deliberation and direction about how the center should function and develop has been an ongoing issue*.

(ST16)

*In my opinion, the central government needs to establish a vision for community-based mental healthcare centers. Simultaneously, clearly defined and achievable objectives are necessary to successfully promote mental healthcare services. As it currently stands, with unclear and unspecified goals, it feels like we are moving forward incrementally, which often leads to intense scrutiny and questioning*.

(ST7)

#### 3.2.2. Failure to Conduct Needs Assessments before Service Delivery

Analysis of the interview data revealed that multiple community-based mental healthcare centers failed to conduct prior investigations and analyses regarding the scope of service delivery or assessments of local resource availability.

*The initial establishment of the center was completed hastily and without proper preparation. We lacked the necessary workforce and expertise to conduct detailed investigations and analyses of the mental health issues and needs of the community members*.

(ST8)

*The development of community-based mental healthcare services should ideally be informed by an understanding of the community’s needs. However, at the time of establishment, there was a lack of analysis regarding the issues and demands of the community*.

(ST12)

#### 3.2.3. Absence of Accessible Direct Mental Healthcare Services

The issue of medical accessibility was brought to attention by international healthcare policy scholars as early as the 1970s, with a focus on the various obstacles that people face when seeking healthcare services. During the interviews, one participant emphasized the importance of community-based mental healthcare centers as follows: “Community-based mental healthcare centers are designed to dispel preconceived stereotypes of mental illnesses and, simultaneously, offer the public tangible mental health promotion services. Their value lies in being accessible, and center locations should be established based on population numbers.” Another interviewee suggested that these centers should be as accessible as convenience stores to encourage public utilization. However, it was noted that the current constraints in staffing and professionalism within these mental healthcare centers hindered their ability to effectively provide accessible direct services.

*Limitations in funding and staffing have hindered our ability to provide direct services effectively, resulting in suboptimal implementation of our services. This, in turn, raises concerns about our ability to meet the public’s demands, leading to criticism of our service quality*.

(ST6)

*Community-based mental healthcare centers are intended to challenge prevailing stereotypes about mental illnesses and provide mental healthcare services and promotions to the public. Their value is rooted in accessibility, with center locations ideally based on population numbers. Unfortunately, we have not fully realized these objectives, which makes us no different from medical clinics*.

(ST11)

*The center is fundamentally unable to provide direct services, leaving referrals the sole available option. This referral process mainly involves providing individuals with information about available resources*.

(ST15)

#### 3.2.4. Limited Service Efficiency

Existing policies mandate the establishment of one community-based mental healthcare center in each county or city. However, these centers were established without considering the vast geographical areas in some counties or cities, some of which encompass as many as 31 townships. This extensive service coverage has resulted in reduced functionality and effectiveness of community-based mental healthcare centers.

*For community-based mental healthcare centers to make significant progress in secondary prevention, the expertise of psychologists is essential. In our county, which has a large population and extensive geographical coverage, addressing the mental health needs of people in townships with just one staff member, alongside a public health nurse, presents a significant challenge*.

(ST1)

*The county’s size and transportation present notable challenges. We have a total of 31 townships, spanning from the mountains to the sea, along with a substantial population. However, our community-based mental healthcare center is located within the county government building, making it relatively unknown to residents in other townships*.

(ST10)

#### 3.2.5. Insufficient Local Mental Healthcare Resources

The interview data demonstrated that local mental healthcare resources were unequally distributed in areas where community-based mental healthcare centers were located. At the time of the study, most mental healthcare resources were concentrated in urbanized regions. Conversely, in rural and remote areas, individuals often could not access proper community-based mental healthcare resources.

*In our county, we only have access to two psychologists, along with two psychiatrists in the Psychiatric Department. In reality, our community-based mental healthcare resources are extremely limited*.

(ST5)

*As an agricultural county, mental healthcare resources are already scarce and insufficient within the county. It is already quite difficult to arrange referrals, not to mention having direct access to mental health professionals due to staffing shortages*.

(ST14)

#### 3.2.6. Stigmatization of Mental Illness

For many years, the Taiwanese government has predominantly employed a medical model to address individuals with mental illnesses. Consequently, society in Taiwan tends to stigmatize mental health issues, leading to significant biases against people facing such challenges. Consequently, when individuals experience psychological distress, they often hesitate to seek assistance from community-based mental healthcare centers due to the fear of potential judgments and prejudices by others.

*People frequently recognize their psychological issues but are hesitant to seek help out of fear. They worry that others might categorize them as having a mental illness and are, therefore, reluctant to seek assistance, dreading potential social stigma and negative perceptions*.

(ST10)

*Negative stereotypes about mental illnesses are prevalent among the general population, with older adults, in particular, exhibiting reluctance to seek support from mental health professionals. This is an area that requires immediate attention*.

(ST6)

#### 3.2.7. Inability to Provide Continuous Mental Healthcare Services

Historically, the care of individuals with mental illnesses predominantly revolved around hospital-based treatment. However, the perspective of community-based mental healthcare centers underscores the significance of consistent and comprehensive services. This approach seeks to establish early symptom detection, direct interventions, follow-up, and case management for individuals, ultimately aiming to facilitate their social reintegration. However, analysis of the interview data revealed that most community-based mental healthcare centers struggled to deliver continuous care services, often due to service fragmentation among various units.

*The absence of service continuity in community mental health is a notable challenge. Presently, community-based mental healthcare centers have yet to address this issue, leading to fragmented mental healthcare services. This problem has persisted over an extended period*.

(ST3)

*It appears that a service platform connecting different departments has yet to be established. The construction of a collaborative service platform is vital, where different disciplines can work closely together. I strongly believe that providing consistent services for individuals with mental illness is crucial, but there are currently significant communication barriers*.

(ST2)

#### 3.2.8. Unable to Provide Case Management

Case management is a method within the social service delivery system that involves coordinating relationships between various service providers and the client to ensure that the latter receives the most suitable and comprehensive care while efficiently utilizing resources. From the interview data, it is evident that community-based mental healthcare centers struggled to effectively implement case management, especially in cases involving suicidal tendencies, mental illness, and other complications.

*We handle numerous cases that require significant attention. Case management is not just a matter of making a phone call; it often entails long-term follow-up and connecting individuals to additional resources*.

(ST9)

*The most significant challenge in managing mental health cases currently is the ability to provide follow-up services for resolving case issues. This is a critical concern because, without the capacity to address case issues, case management is essentially ineffective*.

(ST15)

## 4. Discussion

The findings of this research have revealed that Taiwanese community-based mental healthcare centers face numerous operational challenges. First, these centers struggle with unclear organizational goals and role definitions. In Taiwan, community-based mental healthcare services for the general public have traditionally been offered by psychiatric departments in hospitals and specialized psychiatric hospitals [27,28]. While Taiwan’s Mental Health Act mandates the establishment of community-based mental healthcare centers, their objectives and differentiation from hospital-based psychiatric services have not been clearly defined in relevant policies. Over the past decade, the prevalence of mental disorders in Taiwan has surged, but the budgets and workforce for community-based mental healthcare have not seen proportionate growth [3]. The literature suggests that due to limited budgets and low salaries in the community-based mental healthcare industry, both hospital and mental health professionals are hesitant to transition from hospital-based care [29], contrary to the global shift toward community-based mental healthcare services [30]. Second, the lack of accessible mental healthcare services and insufficient mental healthcare resources in specific regions pose an accessibility challenge. Research findings align with the work of Hiroto et al., who demonstrated that mental healthcare resources in most Asian countries were concentrated in urban areas, leaving resource-deprived rural regions without adequate access to services [31]. Third, persistent stereotypes about mental illness within Taiwan deter individuals from seeking services proactively. In the context of Asian societies, mental illnesses are often associated with malevolent spirits or attributed to personal weaknesses [32]. Additionally, the public’s misconceptions about mental illnesses result in the emergence of prejudice, subsequently leading to discrimination. This results in a significant disparity between national legal safeguards and the social reality, a common issue in many Asian countries [33,34]. Fourth, our research shows that community-based mental healthcare centers struggle to provide continuous mental healthcare services. Long-standing practices of isolating individuals with mental illnesses have led to a gradual deinstitutionalization process in Asian societies. These Asian countries are transitioning from institutional to community care. In contrast to Western countries, Asian nations often express concerns about the potential societal disruption stemming from swift deinstitutionalization. Consequently, they are cautiously decreasing the number of psychiatric beds while making efforts to introduce community-based mental healthcare services. These attempts have faced challenges primarily due to the fragmented nature of mental healthcare systems that arises from the necessity for role differentiation between hospital and community services and the distinction between public and private services [35,36]. Finally, even when individuals can access these mental healthcare services, difficulties may arise during service utilization, primarily because of the absence of needs assessment before service delivery and the extensive service coverage areas.

According to the 2020 Mental Health Atlas published by the World Health Organization (WHO), numerous countries around the world face challenges in achieving the objectives for expanding mental healthcare services [37]. These challenges encompass insufficient government funding for mental health, suboptimal quality of mental healthcare, enduring societal stigma, fear, and shame associated with mental illnesses, as well as issues related to suicide [38,39]. Furthermore, mental healthcare spending in various countries remains relatively low, averaging 2.13% of domestic general government health expenditure. This percentage is notably lower in low-income countries at 1.05%, whereas it is sitting at 3.8% in high-income countries [37]. Globally, the average ratio is approximately 13 mental health workers per 100,000 individuals, with significant disparities between countries. Low-income countries have less than two mental health workers per 100,000 individuals, whereas high-income countries have as many as sixty mental health workers [17]. Additionally, community-based mental healthcare facilities are in short supply, averaging just 0.64 facilities per 100,000 people. Substantial differences exist between urban and rural areas as well as between low- and high-income countries, with low-income countries having only 0.11 units and high-income countries having 5.1 units [40]. Due to many individuals not receiving appropriate treatment for mental illnesses and the substantial demand for community-based mental healthcare services, there exists a treatment gap. Consequently, in 2010, the WHO introduced the Mental Health Gap Action Programme Implementation Guide to assist countries in enhancing their mental health policies [41]. One of the strategies emphasized in this guide is the expansion of community-based mental healthcare resources, with community-based mental healthcare centers playing a pivotal role in addressing these issues.

Nevertheless, as indicated by this study, Taiwan has long been inclined toward a mental healthcare policy that emphasizes psychiatric care, with more resources directed at increasing the capacity of psychiatric beds. Consequently, insufficient resources have been allocated to community-based mental healthcare centers, resulting in an absence of mental healthcare services tailored to the local community’s needs. Moreover, when individuals with mental illnesses are discharged and return to the community, the available mental healthcare resources are inadequate to support their successful reintegration into community life. Burns (2020) identified four key areas that community-based mental healthcare centers should encompass to enhance the mental health of the local population as follows: (1) stressing the provision of services that are accessible and acceptable to the community, (2) harnessing the strengths of individuals with experiences of mental illness, (3) expanding a comprehensive network of support, services, and sufficient resources, and (4) giving priority to evidence-based and recovery-oriented services [42]. In recent years, the Ministry of Health and Welfare has initiated a shift toward developing a community-based mental healthcare service system. Commencing in 2017, the second phase of the national mental healthcare policy has prioritized the establishment of community-based mental healthcare centers for the first time. Local governments have been tasked with establishing one community-based mental healthcare center for every three to four townships, taking into consideration regional geographic characteristics, population distribution, and available mental healthcare resources. These centers aim to provide accessible mental health promotion, counseling, suicide prevention services, and resources. The ultimate objective is to institute a “family-centered, community-based” model for community-based mental healthcare services, effectively overturning the prolonged dominance of psychiatric care in this area.

However, there were several research limitations. First, the present study used convenience sampling, which limits the external validity of the study. Second, the participants in this study were exclusively practitioners from community-based mental healthcare centers, and their viewpoints may not necessarily align with those of policymakers and expert scholars. Hence, future research should expand its scope to encompass policymakers and expert scholars, encouraging a three-way dialogue involving academia, government, and the industry. Third, the sample size was limited, and it suggests that further works should explore the operational status of different community mental healthcare centers. Fourth, this study relied on qualitative research methods to examine the status quo and challenges of community-based mental healthcare center services. Subsequent research can explore the use of quantitative research surveys to collect feedback from the general public to provide an understanding of the public’s perceptions and experiences with community-based mental healthcare services.

## 5. Conclusions

This study has revealed that more than half of the community-based mental healthcare centers in Taiwan have limited functionality and can only provide resource referral services to the public, lacking the capacity to offer comprehensive and accessible direct services. This underscores the need for significant improvements in Taiwan’s community-based mental healthcare service system. To address this issue, a comprehensive system needs to be established, which encompasses primary, secondary, and community-based services, providing a wide range of services to the public, including support, care, recovery, and treatment to meet their mental healthcare service requirements. Furthermore, this calls for flexible and diverse policy development. Based on the findings of this study, the following recommendations are proposed to enhance the current state of community-based mental healthcare center services in Taiwan. First, it is necessary to initiate systematic discussions and consensus building by involving experts, center staff, and community representatives. This collaborative approach can help establish clear standards for constructing the framework and models of community-based mental healthcare centers. In addition, long-term development goals should be customized to suit the specific needs of each region, thereby effectively addressing service challenges. Second, issues related to resource allocation, such as the shortage of professional staff and insufficient budgets, should be overcome. County and city governments should prioritize community-based mental healthcare work, ensuring that centers can operate smoothly with a stable foundation by resolving budget and staffing challenges. Third, the functions of community-based mental healthcare centers should be implemented with a detailed focus on planning service content. Meanwhile, regional differences should be accounted for, and development goals that progressively address the challenges in service delivery should be established. Additionally, community-based mental healthcare work should be promoted with an emphasis on prevention over treatment.

## Figures and Tables

**Table 1:** Demographic characteristics of participants (n = 17).

Characteristics	Categories	N	%
Age (Years) (Range 27–45)	20–29	3	17.6
	30–39	8	47.1
	40–49	6	35.3
Gender	Male	7	41.2
	Female	10	58.8
Education	University	9	52.9
	Master	8	47.1
Professional background	Nursing	7	41.3
	Social work	4	23.5
	Psychology	3	17.6
	Others	3	17.6
Job tenure (Experience) (Range 1–6)	1–3	10	58.8
	4–6	7	41.2

**Table 2:** Summary of themes and subthemes emerging from the interviews.

Theme	Subtheme
1. Operational status	● 1.1 Organizational goals
	● 1.2 Human resources
	● 1.3 Operational budget
	● 1.4 Professional expertise
2. Operational challenges	● 2.1 Lack of clarification in organizational goals and role definition
	● 2.2 Failure to conduct needs assessments before service delivery
	● 2.3 Absence of accessible direct mental healthcare services
	● 2.4 Limited service efficiency
	● 2.5 Insufficient local mental healthcare resources
	● 2.6 Stigmatization of mental illness
	● 2.7 Inability to provide continuous mental healthcare services
	● 2.8 Unable to provide case management

## Data Availability

The data presented in this study are available upon request from the corresponding author.
